# Low-dose aspirin and non-aspirin non-steroidal anti-inflammatory drugs and epithelial ovarian cancer survival: a registry-based cohort study in Norway

**DOI:** 10.1186/s12885-025-14168-y

**Published:** 2025-04-30

**Authors:** Nathalie C. Støer, Edoardo Botteri, Kristina Lindemann, Hilde Langseth, Renée Turzanski Fortner

**Affiliations:** 1 Department of Research, Cancer Registry of Norway, Norwegian Institute of Public Health, Majorstuen 0304, Oslo, 5313 Norway; 2 Section for Colorectal Cancer Screening, Cancer Registry of Norway, Norwegian Institute of Public Health, Oslo, Norway; 3 Department of Gynecological Oncology, Division of Cancer Medicine, Oslo University Hospital, Oslo, Norway; 4 Faculty of Medicine, Institute of Clinical Medicine, University of Oslo, Oslo, Norway; 5 Department of Epidemiology and Biostatistics, School of Public Health, Imperial College London, London, UK; 6 Division of Cancer Epidemiology, German Cancer Research Center, Heidelberg, Germany

**Keywords:** Ovarian cancer, Survival, Aspirin, Non-aspirin NSAIDs

## Abstract

**Background:**

Aspirin and non-aspirin non-steroidal anti-inflammatory drugs (NA-NSAID) have been associated with improved survival in individuals with epithelial ovarian cancer (EOC); however, findings to date are inconsistent.

**Methods:**

We conducted a registry-based cohort study evaluating survival following an incident invasive EOC diagnosis including individuals diagnosed between 2004–2018 (n = 4325; n = 2206 deaths; n = 1973 EOC deaths). Evaluated exposures were low-dose aspirin and NA-NSAIDs. Two primary post-diagnosis exposure windows were evaluated: fixed post-diagnostic baseline exposure ≤ 305 days after diagnosis (use, non-use) and updated “time-varying” exposure (never, past, current use; cumulative defined daily dose (DDD)). Pre-diagnostic exposure (use, non-use) was further evaluated. Multivariable Cox-proportional hazard models were used to estimate hazard ratios (HRs) and 95% confidence intervals [95% CIs]. The primary outcome was cause-specific survival. Restricted mean survival time (RMST) in exposure groups was estimated at 5 years following start of follow-up.

**Results:**

Baseline post-diagnosis aspirin use was not associated with survival following an EOC diagnosis (e.g., use vs. no use: aspirin, HR = 1.02 [95% CI = 0.84–1.24]). Inverse associations were observed between current aspirin use post-diagnosis and survival in the time-varying exposure models (HR 0.68 [0.57–0.81]), and with higher post-diagnosis cumulative DDD of aspirin. Findings for NA-NSAIDs were less consistent. No associations were observed for pre-diagnostic use. Results for overall survival were similar to those for cause-specific survival. Compared to never use, post-diagnosis low-dose aspirin use was associated with a longer RMST (e.g., ever vs. never use, difference in RMST = 2.67 months).

**Conclusions:**

This study provides further evidence of a potential beneficial effect of post-diagnosis low-dose aspirin use for ovarian cancer survival.

**Supplementary Information:**

The online version contains supplementary material available at 10.1186/s12885-025-14168-y.

## Introduction

Epithelial ovarian cancer (EOC) remains the most lethal gynecologic malignancy, with relatively poor survival even in the context of optimal debulking surgery and recent advancements in treatment options [1, 2]. There are relatively few modifiable factors associated with improved survival [3], with common medication use an area of active investigation with respect to EOC outcomes [4].

Aspirin in higher doses (i.e., ≥ 300 mg) and non-aspirin non-steroidal anti-inflammatory drugs (NA-NSAIDs) may impact cancer survival given their anti-inflammatory properties mediated via cyclooxygenase-1 and − 2 (COX-1 and − 2) inhibition [5, 6]. Lower dose aspirin inhibits COX-1, which is implicated in control of platelet activation, a factor associated with cancer promotion, progression, metastasis, and angiogenesis [6, 7]. Aberrant platelet activation is implicated in EOC progression [8]. Post-diagnosis aspirin and NA-NSAID use have been associated with improved survival in women with a diagnosis of EOC [9–11], though results are not consistent [12]. Similarly, findings on aspirin and NA-NSAID use prior to diagnosis and cancer outcomes are equivocal [9–13]. Prior studies have predominantly evaluated self-reported exposure [9, 10, 13] with variable definitions of exposure, and with individual registry-based studies on aspirin [12] and NA-NSAIDs [11] and EOC survival from Denmark.

Given the strong biologic plausibility and relatively sparse and inconsistent data to date, our primary aim was to evaluate associations between use of prescribed low-dose aspirin and NA-NSAIDs, and survival following an EOC diagnosis with a primary focus on post-diagnosis use.

## Methods

### Study population

Invasive EOC cases were identified through the Cancer Registry of Norway (CRN). The CRN was established in 1951 with mandatory reporting since 1953. The coding system follows international standards [14]; completeness is estimated to be 98.6% [15]. Tumors are classified according to ICD-O-3 (International Classification of Diseases for Oncology 3rd edition). The Norwegian Prescription Database (NorPD) was established in 2004; cases diagnosed from June 2004 were eligible for inclusion. Immigration data were available from the Central Population Register, and mortality data including cause of death from the Norwegian Cause of Death Registry. The full cohort includes 1.8 million women, ages 18–79 years with follow up from 2004 to 2018.

A total of 5370 women with incident invasive EOC (ICD10 = C56, C57.0-4 or C48.2) as their first cancer diagnosis (except for non-melanoma skin cancer ICD10 = C44) and born between 1925 and 1986 were identified as potentially eligible. Women not residing in Norway (*n* = 28) or diagnosed in the 6 months after immigration to Norway (*n* = 7; pre-diagnosis use not available/complete) were excluded, together with 1010 individuals surviving < 10 months following diagnosis. A total of 4325 EOC cases were included, with 2206 deaths observed (*n* = 1973 cause-specific) over follow-up. Individuals were followed-up until death, emigration or December 31, 2018, whichever occurred first.

### Exposure and covariate data

NorPD includes all prescription medication for individuals in Norway including date of dispensing and medications dispensed (identified by Anatomical Therapeutic Chemical (ATC) code), strength (i.e., active ingredient per unit, e.g., mg per tablet), and defined daily dose (DDD) units (average maintenance dose per day for a medication for its main indication).

Aspirin and NA-NSAIDs were identified via ATC code. Prescription aspirin was 75 mg (82%) and 160 mg (13%) doses (ATC: B01AC06) with a small proportion (5%) dipyridamole (200 mg) and aspirin (25 mg) (B01AC30); thus, only lower-dose aspirin was considered. NA-NSAIDs included were predominantly ibuprofen (76.9% of women with a NA-NSAID prescription) and diclofenac (61.9% with a prescription). Supplemental Table 1 includes the ATC codes for included formulations, percent of the drug class with a prescription for the individual drug, and first and last dates of use observed.

NorPD was also used to identify medications modelled as confounders as proxies for their indication: pre- and post-diagnosis statins (C10), anti-diabetics (A10), and cardiovascular drugs (C01-C09). Further covariates, including number of children and age at first birth, income, education, and immigration background were obtained via a linkage with Statistics Norway. Details about the cancer diagnosis was available from the CRN incidence registry.

### Statistical analyses

The main outcome was cause-specific survival. Overall survival was evaluated in a secondary analysis. Multivariable Cox-proportional hazard models, with time since diagnosis as the time-scale, were used to model survival, controlling for age at diagnosis (continuous), histology groups (for models including all cases; high-grade serous, low-grade serous, endometrioid, mucinous, clear cell, carcinosarcoma; ICD-O-3 codes in Supplemental Table 2), stage (localized, regional, distant, missing), ethnicity (Norway, other Nordic countries, other), education (mandatory level, secondary, higher education, missing), marital status (single, married/partnered, widowed/separated/divorced), and use of other medications at baseline (medications indicated for cardiovascular disease, statins and anti-diabetics).

**Fig. 1:**
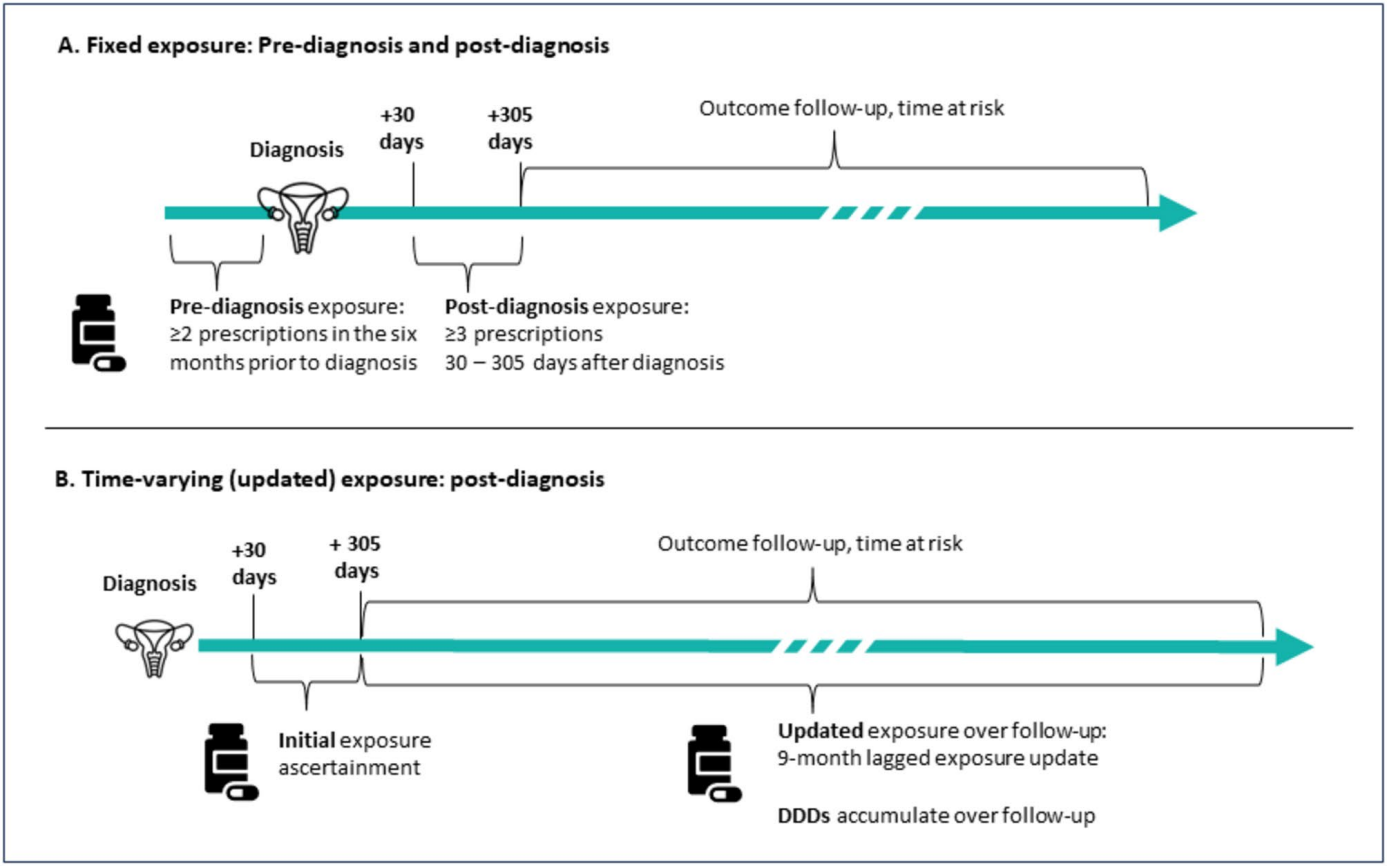
Exposure was modeled considering (**A**) Fixed exposure (use or non-use) both pre-diagnosis (≤ 6 months prior to diagnosis) and baseline post-diagnosis (> 30 days and ≤ 305 days (10 months) following diagnosis); and (**B**) updated post-diagnosis exposure over follow-up considering never, current, and past use and updated cumulative defined daily dose (DDD) with a nine-month lagged exposure update introduced to reduce the effect of reverse causation

Pre-diagnostic use was defined as ≥ 2 prescriptions in the six months prior to diagnosis. For post-diagnosis use, the exposure period started 30 days after diagnosis, and we evaluated both a fixed exposure, considering baseline exposure only (Fig. 1A) and a time-varying exposure with updated exposure over follow-up (Fig. 1B). For post-diagnosis baseline exposure, exposed women had ≥ 3 filled prescriptions between 30- and 305 days following diagnosis; the remainder of the study population were classified as unexposed. Three filled prescriptions was selected to ensure regular use, under the assumption that those who refilled a prescription twice were more likely to represent regular users than those with fewer refills. We evaluated one or two filled prescriptions in a sensitivity analysis. Time at risk started at 305 days following diagnosis for all analyses; in a sensitivity analysis for pre-diagnosis exposure, time at risk started at diagnosis.

In the post-diagnosis time-varying exposure model, we evaluated current, past, ever, and never exposure and further evaluated cumulative DDD. Women were classified as “unexposed” until three consecutive prescriptions were filled, after which they were “current” users if they continued to fill prescriptions or 4 months following the last prescription. Individuals were classified as “past” users > 4 months following the last prescription and could be re-classified as “current” users over follow-up. Individuals were classified as an “ever” user when classified as either a “past” or “current” user. Cumulative DDD was updated at each new prescription. Analyses considering updated exposure included a nine-month lagged exposure update to reduce the effect of reverse causation (e.g., those with disease progression may cease use of these drugs). In a sensitivity analysis to further address reverse causation, we evaluated a more conservative two-year lagged exposure update (*n* = 3095 individuals). Analyses were also conducted restricted to cases with known high-grade serous EOC, and individuals with metastatic disease at diagnosis.

Flexible parametric survival models with restricted cubic splines with 5 degrees of freedom for the log cumulative hazard were used to estimate standardized survival curves and adjusted restricted mean survival time (RMST) at 5 years following the start of follow-up. The flexible parametric models were adjusted the same way as the Cox-regression models, and post-diagnosis aspirin use “ever” vs. “never”, and “current” and “past” vs. “never” use were compared. The RMST estimates survival time at a fixed time point, here at 5 years after the start of follow-up, and is the observed survival time for all women with a survival time ≤ 5 years and set to 5 years for all women with a survival time > 5 years. The difference in RMST between exposure groups provides an estimate of the difference in mean survival time associated with exposure.

All tests were two-sided. Statistical analyses were performed using R version 4.2.3 (http://cran.r-project.org/). Standardized survival curves and RMST were estimated using stpm3 in Stata version 18.0.

## Results

Relative to women classified as non-users of either aspirin or NA-NSAIDs, women classified as aspirin users at study baseline (i.e., beginning of follow-up) were older at diagnosis (median age, users = 71.0 years, non-users = 62.9), less likely to be married (users = 49.4%, non-users = 57.7%), nulliparous (users = 10.0%; non-users = 15.3%), or with education beyond the level of mandatory school (users = 63.1%; non-users = 72.2%) (Table 1). Baseline characteristics of women classified as NA-NSAID users were similar to those of non-users. The majority of cases were of serous high-grade (46.3%) or unknown grade (30.4%) histology, with metastatic disease at diagnosis (72.6%). A total of 49.0% of individuals were alive at the end of follow-up, 45.6% died of ovarian cancer, and 5.4% died of another cause. Median follow-up time was 2.6 years (maximum = 13.6).

**Table 1:** Baseline characteristics at beginning of follow-up: Registry-based cohort of epithelial ovarian cancers diagnosed in Norway 2004–2018

	No use (*N* = 3627)	Aspirin only (*N* = 249)	Non-Aspirin NSAIDs only (*N* = 431)	Both (*N* = 18)	Total (*N* = 4325)
**Age at diagnosis**					
Median (Q1, Q3)	62.9 (54.7, 70.7)	71.0 (65.6, 78.2)	60.4 (53.5, 67.3)	69.2 (63.7, 73.1)	63.2 (55.1, 70.9)
**Health region**					
South-East	2074 (57.2%)	129 (51.8%)	316 (73.3%)	14 (77.8%)	2533 (58.6%)
West	694 (19.1%)	61 (24.5%)	70 (16.2%)	1 (5.6%)	826 (19.1%)
Mid	495 (13.6%)	31 (12.4%)	19 (4.4%)	2 (11.1%)	547 (12.6%)
North	364 (10.0%)	28 (11.2%)	26 (6.0%)	1 (5.6%)	419 (9.7%)
**Ethnicity**					
Norway	3319 (91.5%)	239 (96.0%)	391 (90.7%)	17 (94.4%)	3966 (91.7%)
Other Nordic	88 (2.4%)	5 (2.0%)	11 (2.6%)	0 (0.0%)	104 (2.4%)
Rest of the world	220 (6.1%)	5 (2.0%)	29 (6.7%)	1 (5.6%)	255 (5.9%)
**Marital status**					
Single	439 (12.1%)	19 (7.6%)	44 (10.2%)	0 (0.0%)	502 (11.6%)
Married/partnered	2093 (57.7%)	123 (49.4%)	239 (55.5%)	11 (61.1%)	2466 (57.0%)
Widowed/separated/divorced	1095 (30.2%)	107 (43.0%)	148 (34.3%)	7 (38.9%)	1357 (31.4%)
**Children**					
0	554 (15.3%)	25 (10.0%)	59 (13.7%)	1 (5.6%)	639 (14.8%)
1	554 (15.3%)	33 (13.3%)	59 (13.7%)	1 (5.6%)	647 (15.0%)
2	1345 (37.1%)	97 (39.0%)	162 (37.6%)	9 (50.0%)	1613 (37.3%)
3	807 (22.2%)	57 (22.9%)	111 (25.8%)	4 (22.2%)	979 (22.6%)
4+	367 (10.1%)	37 (14.9%)	40 (9.3%)	3 (16.7%)	447 (10.3%)
**Age at first birth**					
Nulliparous	554 (15.3%)	25 (10.0%)	59 (13.7%)	1 (5.6%)	639 (14.8%)
<25	1763 (48.6%)	141 (56.6%)	215 (49.9%)	15 (83.3%)	2134 (49.3%)
25–29	876 (24.2%)	54 (21.7%)	109 (25.3%)	1 (5.6%)	1040 (24.0%)
30+	434 (12.0%)	29 (11.6%)	48 (11.1%)	1 (5.6%)	512 (11.8%)
**Education**					
Non/mandatory only	973 (26.8%)	90 (36.1%)	131 (30.4%)	7 (38.9%)	1201 (27.8%)
Secondary	1688 (46.5%)	125 (50.2%)	199 (46.2%)	10 (55.6%)	2022 (46.8%)
Higher	934 (25.8%)	32 (12.9%)	100 (23.2%)	1 (5.6%)	1067 (24.7%)
Missing	32 (0.9%)	2 (0.8%)	1 (0.2%)	0 (0.0%)	35 (0.8%)
**Cardiovascular drugs**					
No	2504 (69.0%)	73 (29.3%)	314 (72.9%)	3 (16.7%)	2894 (66.9%)
Yes	1123 (31.0%)	176 (70.7%)	117 (27.1%)	15 (83.3%)	1431 (33.1%)
**Statins**					
No	3003 (82.8%)	97 (39.0%)	360 (83.5%)	9 (50.0%)	3469 (80.2%)
Yes	624 (17.2%)	152 (61.0%)	71 (16.5%)	9 (50.0%)	856 (19.8%)
**Anti-diabetics**					
No	3456 (95.3%)	219 (88.0%)	414 (96.1%)	16 (88.9%)	4105 (94.9%)
Yes	171 (4.7%)	30 (12.0%)	17 (3.9%)	2 (11.1%)	220 (5.1%)
**SEER stage**					
Localized	757 (20.9%)	35 (14.1%)	77 (17.9%)	2 (11.1%)	871 (20.1%)
Regional	144 (4.0%)	17 (6.8%)	12 (2.8%)	0 (0.0%)	173 (4.0%)
Metastatic	2613 (72.0%)	185 (74.3%)	326 (75.6%)	15 (83.3%)	3139 (72.6%)
Missing	113 (3.1%)	12 (4.8%)	16 (3.7%)	1 (5.6%)	142 (3.3%)
**Histology**					
High-grade serous	1657 (45.7%)	109 (43.8%)	230 (53.4%)	7 (38.9%)	2003 (46.3%)
Low-grade serous	176 (4.9%)	12 (4.8%)	24 (5.6%)	1 (5.6%)	213 (4.9%)
Serous unknown grade	1116 (30.8%)	88 (35.3%)	102 (23.7%)	10 (55.6%)	1316 (30.4%)
Endometroid	211 (5.8%)	9 (3.6%)	24 (5.6%)	0 (0.0%)	244 (5.6%)
Mucinous	220 (6.1%)	16 (6.4%)	22 (5.1%)	0 (0.0%)	258 (6.0%)
Clear cell	185 (5.1%)	9 (3.6%)	24 (5.6%)	0 (0.0%)	218 (5.0%)
Carcinosarcoma	62 (1.7%)	6 (2.4%)	5 (1.2%)	0 (0.0%)	73 (1.7%)

### Low-dose aspirin

Pre-diagnosis low-dose aspirin exposure was not associated with cause-specific survival (HR = 1.08 [95% CI: 0.92–1.26] and no associations were observed for post-diagnosis baseline use (HR = 1.02 [0.84–1.24]) (Table 2).

We next evaluated outcomes considering updated exposure over follow-up. Current low-dose aspirin use was associated with better cause-specific survival (HR = 0.68 [0.57–0.81]), as compared to non-use. Similarly, “ever” post-diagnosis use was associated with better survival (HR = 0.76 [0.64–0.89]), whereas no association was observed for past users (HR = 1.25 [0.91–1.70]). Higher cumulative aspirin DDD post-diagnosis was associated with better survival (compared to non-users, DDD < median, HR = 0.85 [0.71–1.03]; ≥median, HR = 0.57 [0.42–0.76]).

### NA-NSAID use

We observed no associations between pre-diagnosis or post-diagnosis baseline use of NA-NSAIDs and cause-specific survival (pre-diagnosis, HR = 1.10 [0.93–1.30]; post-diagnosis baseline, HR = 1.11 [0.92–1.34]) (Table 3).

We observed divergent associations for current and past NA-NSAID use when evaluating updated exposure over follow-up. Compared to non-users, current use was not associated with survival (HR = 0.92 [0.74–1.14]), while past use was associated with worse survival (HR = 1.20 [1.05–1.38]). Worse survival was also observed with cumulative DDD above the median (HR = 1.27 [1.09–1.49]).

**Table 2:** Pre- and post-diagnosis low-dose aspirin use and cause-specific survival following an ovarian cancer diagnosis: epithelial ovarian cancers diagnosed in Norway 2004–2018

Timing of exposure and exposure level	Deaths	Person-years	HR^a^		95% CI	
**Pre-diagnosis**						
No aspirin	1774	15,088	Ref.			
Aspirin use	199	1210	1.08	0.92	-	1.26
**Post-diagnosis**,** Baseline exposure**						
No aspirin	1843	15,431	Ref.			
Aspirin use	130	867	1.02	0.84	-	1.24
Only post	49	341	0.97	0.72	-	1.30
Pre and post	81	526	1.06	0.84	-	1.33
**Post-diagnosis**,** Updated exposure**						
No aspirin	1789	14,336	Ref.			
Ever aspirin	184	1968	0.76	0.64	-	0.89
Current aspirin	141	1663	0.68	0.57	-	0.81
Past aspirin	43	305	1.25	0.91	-	1.70
No aspirin	1789	14,336	Ref.			
DDD < median	132	944	0.85	0.71	-	1.03
DDD ≥ median	52	1024	0.57	0.42	-	0.76

^a^Multivariable models controlling for age at diagnosis (continuous), histology groups (high grade serous, low grade serous, endometrioid, mucinous, clear cell, carcinosarcoma), stage (localized, regional, distant, missing), ethnicity (Norway, other Nordic, other), education (mandatory level, secondary, higher education, missing), marital status (single, married/partnered, widowed/separated/divorced), and use of other medications at baseline (medications indicated for cardiovascular disease, and statins and anti-diabetics) Abbreviations: DDD = Defined Daily Dose; NSAIDs = non-steroidal anti-inflammatory drugs; NA-NSAIDs = non-aspirin NSAIDs

**Table 3:** Pre- and post-diagnosis NA-NSAID use and cause-specific survival following an ovarian cancer diagnosis: epithelial ovarian cancers diagnosed in Norway 2004–2018

Timing of exposure and exposure level	Deaths	Person-years	HR^a^	95% CI		
**Pre-diagnosis**						
No NA-NSAIDs	1812	15,068	Ref.			
NA-NSAIDs use	161	1230	1.10	0.93	-	1.30
**Post-diagnosis**,** Baseline exposure**						
No NA-NSAIDs	1733	14,452	Ref.			
NA-NSAIDs use	240	1846	1.09	0.92	-	1.29
Only post	185	1386	1.11	0.92	-	1.33
Pre and post	55	460	1.05	0.79	-	1.40
**Post-diagnosis**,** Updated exposure**						
No NA-NSAIDs	1483	11,741	Ref.			
Ever NA-NSAIDs	490	4563	1.13	1.00	-	1.28
Current NA-NSAIDs	96	944	0.92	0.74	-	1.14
Past NA-NSAIDs	394	3619	1.20	1.05	-	1.38
No NA-NSAIDs	1483	11,741	Ref.			
DDD < median	252	2451	1.03	0.89	-	1.19
DDD ≥ median	238	2111	1.27	1.09	-	1.49

^a^Multivariable models controlling for age at diagnosis (continuous), histology groups (; high grade serous, low grade serous, endometrioid, mucinous, clear cell, carcinosarcoma), stage (localized, regional, distant, missing), ethnicity (Norway, other Nordic, other), education (mandatory level, secondary, higher education, missing), marital status (single, married/partnered, widowed/separated/divorced), and use of other medications at baseline (medications indicated for cardiovascular disease, and statins and anti-diabetics)

Abbreviations: DDD = Defined Daily Dose; NSAIDs = non-steroidal anti-inflammatory drugs; NA-NSAIDs = non-aspirin NSAIDs

### Overall survival and RMST

**Fig. 2:**
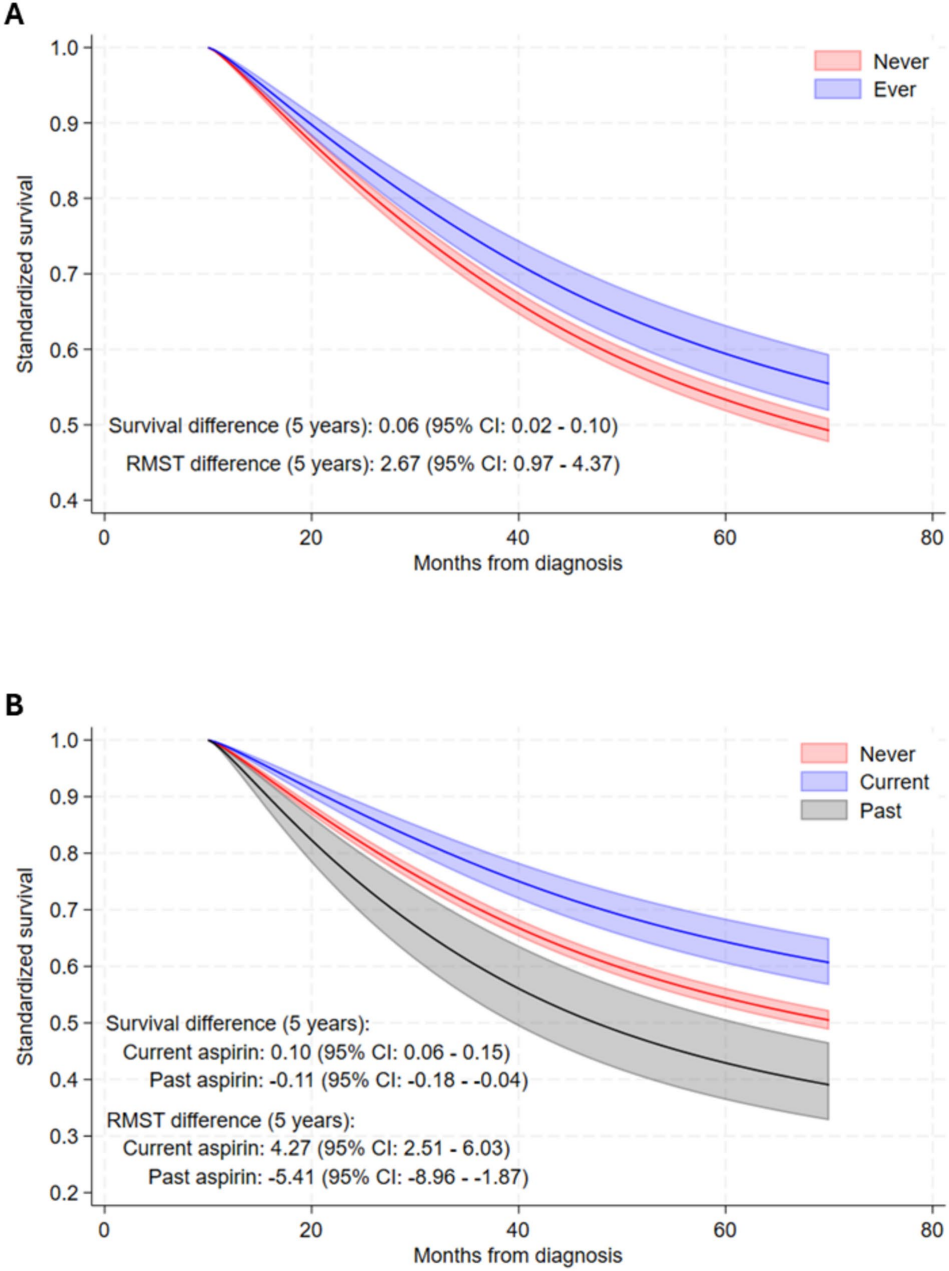
Survival curves for: (**A**) ever and never use of post-diagnosis low-dose aspirin and (**B**) current, past and never use of post-diagnosis aspirin, with estimated restricted mean survival time (RMST) at 5-years after the start of follow-up. The survival curves and RMST were adjusted using regression standardization (i.e. one survival curve for each level of the exposure for each individual was predicted from the model using the observed covariates and the given exposure level, and then averaged at each exposure level). The difference in RMST provides an estimate of the difference in survival time at 5-years after the start of follow-up associated with exposure levels

Results for aspirin (Supplemental Table 3) and NA-NSAIDs (Supplemental Table 4) were similar when evaluating overall survival (89% of deaths were due to EOC). We estimated the RMST as an estimate of the difference in overall survival time by exposure status at 5 years following the start of follow-up. Individuals classified as ever low-dose aspirin user had mean 2.67 (95% CI: 0.97–4.37) months longer survival at that time point, relative to never users (Fig. 2A). Current aspirin use, relative to never use, was associated with 4.27 (95%CI: 2.51–6.03) months longer survival at 5 years following the start of follow-up (Fig. 2B). Past use was associated with worse survival (-5.41 (95% CI: -8.86 to -1.97) months).

### Sensitivity analyses

Results for pre-diagnosis aspirin (HR = 0.98 [0.86–1.13]) and NA-NSAIDs (HR = 1.13 [0.98–1.30]) were not meaningfully different from the primary analysis when follow-up started at diagnosis. Findings for baseline exposure were similar when evaluating one or two prescriptions filled during the baseline post-diagnosis period (Supplemental Table 5). We evaluated updated post-diagnosis exposure using a two-year lagged exposure update (Supplemental Table 6) observing results similar to those using the nine-month lagged exposure update, with the exception of NA-NSAID current use, which was significantly associated with survival in the two-year lagged analysis, but not in the primary analysis (two-year lag, HR = 0.66 [0.48–0.90]; nine-month lag, HR = 0.92 [0.74–1.14]).

Associations were comparable when restricted to high-grade serous cases and when restricted to individuals with metastatic disease at diagnosis for both low-dose aspirin (Supplemental Table 7) and NA-NSAIDs (Supplemental Table 8).

## Discussion

This large registry-based cohort study is one of the largest studies to date and provides further evidence of a potential beneficial effect of aspirin use for ovarian cancer survival. Results were less clear for NA-NSAIDs, but overall provided no evidence of a protective effect of pre- or post-diagnosis use.

Low-dose aspirin use was associated with lower risk of death following an EOC diagnosis in our analyses considering updated exposure over follow-up. We observed 32% lower risk of EOC death comparing current vs. never users (24% for ever users), and 43% lower risk comparing those above the median DDD to non-users. These results provide additional evidence for a beneficial effect of low-dose aspirin after an EOC diagnosis, are in line with previous observations in the Nurses’ Health Study (NHS) and NHSII [9], where a 32% lower risk of death was reported for current post-diagnosis use (in the past 2 years) relative to never use, and the recent findings from the Ovarian cancer Prognosis And Lifestyle (OPAL) cohort where a 35% lower risk of death was observed for ≥ 4 days /week NSAID (aspirin or non-aspirin) use vs. non- or occasional users. No association was observed in a registry-based study in Denmark, which evaluated no post-diagnosis use as compared to ≥ 1 post-diagnosis prescription(s). The differing findings in the registry-based study from Denmark and the current study, may be in part explained by differences in exposure definition. The requirement of at least three consecutive filled prescriptions to be considered “exposed” in the current study was implemented to capture longer-term use, in contrast to the use of ≥ 1 prescription used in the Danish study, which would capture sporadic and regular use. Prior findings on pre-diagnosis use are inconsistent. We observed no association for pre-diagnosis use, in line with the majority of prior studies [9, 12, 13]. Inverse associations were observed in the OPAL study [16] and in a sensitivity analysis in the Ovarian Cancer Association Consortium (OCAC) comparing regular use to use less than once per week (no association observed in primary analysis) [13]; both studies evaluated overall NSAID use as the main exposure.

We evaluated differences in RMST for aspirin exposure levels, given the consistently observed inverse associations. Mean survival time at 5-years following the start of follow-up was longer for ever (difference = 2.67 months) and current users (difference = 4.37 months), compared to never users. These findings are in line with recent observations from the OPAL study, which reported a 2.5-month difference in mean survival time at 5 years post-diagnosis, though this was for aspirin and NA-NSAID use combined. The RMST findings from the current study reflect overall survival, and not cause-specific survival, though 89% of the deaths were due to EOC. Further, while we observed longer survival for both ever and current aspirin use, it is notable that past use was associated with shorter survival in this study. There were a limited number of overall deaths (*n* = 63) in the past users, and we did not observe statistically significant associations for past use in the analysis of cause-specific survival. Nonetheless, reverse causation cannot be excluded.

Post-diagnosis current NA-NSAID use was not associated with better survival. Worse survival was observed in the analysis considering cumulative dose (DDD) and past use. Prior studies on NA-NSAID use and survival following an EOC diagnosis have yielded mixed results, with inverse associations observed for current use in the NHS/II [9] and higher cumulative or intensity use in a registry study in Denmark [11], and the previously described results from the OPAL study on NSAID use overall. The results on NA-NSAIDs in this study should be interpreted with caution, given past use was associated with higher risk of death in this analysis which may be indicative of reverse causation (i.e., cessation with disease progression and use maintained only in healthier individuals). Notably, discontinuation of NA-NSAID use (i.e., past use) was far more common than discontinuation of aspirin use (percent of person-time among ever users that was past use: NA-NSAIDs = 79%; aspirin = 15%). We observed no association between pre-diagnosis use and survival, in agreement with some [9, 11], but not all [13], prior studies. The current study does not provide evidence in support of a beneficial effect of NA-NSAID use for risk of death following an EOC diagnosis.

NSAIDs (including aspirin) have well-described analgesic, anti-pyretic, and anti-inflammatory properties. The canonical actions of NSAIDs are mediated through COX-1 and COX-2 inhibition, and subsequent effects on prostaglandin release. COX-1 regulates basal prostaglandin release, whereas COX-2 is largely recognized for its role in prostaglandin release in response to injury, infection, or a developing neoplasm [6]. In our analyses on aspirin, we evaluated low-dose aspirin, the prescribed formulation in Norway. The effects of the lower-dose aspirin considered in this study are most likely through actions as a selective inhibitor of platelet-derived COX-1 [6, 17] (not reaching the threshold necessary for COX-2 activation) and through potential effects on platelet biology [6]. Low-dose aspirin has well-described anti-platelet effects via COX-1, reducing prostaglandin production and thromboxane A2 (TXA_2_) and TXA_2_-mediated platelet aggregation [7]. Platelets have a well-established role in the development of the (pre-)neoplastic niche, as well as in tumor growth and survival of circulating tumor cells [8]. Individual NA-NSAIDs have differing levels of inhibition of COX-1 and COX-2 [18]. For example, ibuprofen, the mostly commonly prescribed NA-NSAID in this study, has demonstrated somewhat higher levels of COX-1 vs. COX-2 inhibition at therapeutic levels, whereas other NA-NSAIDs, including diclofenac, the second most frequently prescribed NA-NSAID in the study, demonstrate higher levels of inhibition of COX-2 vs. COX-1 [18]. Beyond platelet-mediated actions, the hypothesized mechanisms for NA-NSAIDs in this study included anti-inflammatory effects via COX-1 and − 2 inhibition, and subsequent inhibition of prostaglandin generation from arachidonic acid, as well as COX-independent pathways associated cancer progression [19].

While aspirin and NA-NSAIDs share a mechanistic pathway via COX inhibition, the longer-term sequela of these drugs on COX inhibition may differ. For example, aspirin inhibition of platelet COX-1 is not reversible [20], with a durable effect for the lifespan of the affected platelets (7–10 days) [21]. Given that low-dose aspirin would be expected to be used consistently and not sporadically, this would result in persistent long-term COX-1 inhibition. In contrast, the reversibility of COX inhibition varies by NA-NSAID: rapid but reversible inhibition is noted for ibuprofen, slow and non-reversible effects are reported for diclofenac, and slow and reversable effects are reported for naproxen [22]. These differential effects for low-dose aspirin and NA-NSAIDs, together with differing patterns of use (i.e., more persistent use of low-dose aspirin prescribed for cardiovascular protection) may account for the differences in associations observed for low-dose aspirin and NA-NSAIDs in this study.

Aspirin has been associated with lower cancer risk (e.g., cancers of the breast, colorectum, gastric cancers) [23], including EOC [24, 25], and lower risk of death following a cancer diagnosis (e.g., cancers of the breast, colorectum, composite group of cancer sites) [26], though results are not fully consistent. The protective associations observed with cancer are balanced with associations with gastrointestinal bleeding, ulcer, and hemorrhagic stroke with long-term use [27]. Associations for NA-NSAIDs differ by cancer site and subtype, e.g., with differing results for breast cancer by hormone receptor status (reviewed in [28]), no association observed in a meta-analysis on endometrial cancer [29], and a positive association observed for kidney cancer (reviewed in [30]). Potential risks associated with NA-NSAID use differ by formulation, but include gastrointestinal bleeding and cardiovascular events [31].

Our study has notable strengths and limitations. First, we evaluated prescription aspirin and NA-NSAIDs in an unselected population-based cohort. A strength of the study was the availability of high-quality registry data; however, as with other registry-based studies we have data on prescriptions filled but not on use or compliance. We defined our primary exposure as at least 3 filled prescriptions under the assumption that individuals repeatedly (re-)filling their prescriptions would be most likely to be habitual users. As with other studies some “non”-users in the current study are likely sporadic users. Low-dose aspirin and some NA-NSAIDs are available over-the-counter (OTC) and this use was not captured in our study. However, individuals regularly taking low-dose aspirin or NA-NSAIDs would be expected to have a prescription for this medication given the limited availability, cost, and tight regulation of OTC analgesics. Further, we were only able to capture prescriptions dispensed at the pharmacy and not medication given in a hospital or retirement home. However, there is notable agreement between our findings on aspirin and those using self-reported data [9, 10], which are subject to other limitations. While we had data on case characteristics such as histotype and stage, we did not have data on prognostic factors such as BRCA-mutation status or outcomes following surgery. Finally, while we have used a conservative approach when evaluating updated exposure, implementing lagged update of exposure, we cannot rule out reverse causation.

## Conclusions

We observed inverse associations for post-diagnosis aspirin use and lower risk of death among a population of women diagnosed with EOC, with consistent association for high-grade serous disease and among patients with metastatic disease at diagnosis. Findings were not consistent for NA-NSAIDs. These findings provide further evidence that low-dose aspirin may confer a survival benefit, consistent with some prior studies. Further studies are needed to evaluate the overall risks and benefits of low-dose aspirin use among individuals with an EOC diagnosis.

## Electronic supplementary material

Below is the link to the electronic supplementary material.

Supplementary Material 1

## Data Availability

The data used in this study area are available following ethical approval by application to helsedata.no.

## Abbreviations

ATC: Anatomical therapeutic chemical
CI: Confidence interval
COX: Cyclooxygenase
CRN: Cancer Registry of Norway
DDD: Defined daily dose
EOC: Epithelial ovarian cancer
HR: Hazard ratio
ICD-O: International classification of diseases for oncology
NA-NSAID: Non-aspirin non-steroidal anti-inflammatory drug
NorPD: Norwegian prescription database
OCAC: Ovarian Cancer Association Consortium
OPAL: Ovarian cancer prognosis and lifestyle
OTC: Over the counter
RMST: Restricted mean survival time
TXA_2_: Thromboxane A2
