# Supplementary material for: Low-dose aspirin and non-aspirin non-steroidal anti-inflammatory drugs and epithelial ovarian cancer survival: a registry-based cohort study in Norway
Source: BMC Cancer. 2025 Apr 30;25:807. doi: 10.1186/s12885-025-14168-y (PMC12042365; doi:10.1186/s12885-025-14168-y)
Supplement: Supplementary file 1 — Supplementary Material 1 [file 12885_2025_14168_MOESM1_ESM.docx]

| **Aspirin** | | | | | | | |
| --- | --- | --- | --- | --- | --- | --- | --- |
| **ATC Code** | **Name** | **DDD** | **Individuals** | **Among exposed, % exposed to formulation** | **Earliest prescription in study** | **Latest prescription in study** | **Median DDD per prescription** |
| B01AC06 | acetylsalicylic acid | 1 ED | 693 | 96.9 | 16.09.2004 | 16.12.2018 | 100 |
| B01AC30 | dipyridamol/acetylsalicylic acid | 2 ED | 37 | 5.2 | 16.08.2008 | 16.12.2018 | 14 |
|  |  |  |  |  |  |  |  |
| **NA-NSAIDs** | | | | | | | |
| **ATC Code** | **Name** | **DDD** | **Individuals** | **Among exposed, % exposed to formulation** | **Earliest prescription in study** | **Latest prescription in study** | **Median DDD per prescription** |
| M01AB05 | diclofenac | 0.1 g | 868 | 61.9 | 16.09.2004 | 16.12.2018 | 10 |
| M01AC01 | piroxicam | 0.02 g | 115 | 8.2 | 16.12.2004 | 16.11.2018 | 20 |
| M01AC06 | meloxicam | 15 mg | 30 | 2.1 | 16.03.2006 | 16.12.2018 | 50 |
| M01AE01 | ibuprofen | 1.2 g | 1079 | 76.9 | 16.08.2004 | 16.12.2018 | 33 |
| M01AE02 | naproxen | 0.5 g | 271 | 19.3 | 16.08.2004 | 16.12.2018 | 50 |
| M01AE03 | ketoprofen | 0.15 g | 20 | 1.4 | 16.09.2005 | 16.10.2018 | 67 |
| M01AH01 | celecoxib | 0.2 g | 112 | 8.0 | 16.12.2004 | 16.11.2018 | 30 |
| M01AH04 | parecoxib | 0.04 g | 1 | 0.1 | 16.10.2014 | 16.10.2014 | 10 |
| M01AH05 | etoricoxib | 0.06 g | 166 | 11.8 | 16.12.2005 | 16.12.2018 | 28 |
| M01AX01 | nabumetone | 1 g | 21 | 1.5 | 16.10.2004 | 16.11.2018 | 50 |
| M01AX05 | glucosamine | 1.5 g | 116 | 8.3 | 16.07.2005 | 16.12.2018 | 81 |
| DDD = Defined Daily Dose | | | | | | | |

**Supplemental Table 1.** Anatomical Therapeutic Chemical (ATC) Codes of evaluated aspirin and non-aspirin non-steroidal anti-inflammatory drugs (NA-NSAID) formulations

| **Supplemental Table 2. Morphology ICD03 codes included within each histology group. Cases diagnosed during 2004-2018.** | | | | | |
| --- | --- | --- | --- | --- | --- |
| **High-grade Serous** | **Low-grade Serous** | **Endometroid** | **Mucinous** | **Clear Cell** | **Carcinosarcoma** |
| 8020/3* | 8441/3 | 8380/3 | 8470/3 | 8310/3 | 8951/3 |
| 8140/3* | 8460/3 | 8560/3 | 8474/3 |  | 8980/3 |
| 8255/3* |  | 8570/3 | 8480/3 |  |  |
| 8260/3 |  |  |  |  |  |
| 8380/3* |  |  |  |  |  |
| 8441/3 |  |  |  |  |  |
| 8450/3 |  |  |  |  |  |
| 8460/3 |  |  |  |  |  |
| 8461/3 |  |  |  |  |  |
| ICD = International Classification of Diseases | | | | | |

| **Supplemental Table 3.** Pre- and post-diagnosis aspirin use and overall survival following an ovarian cancer diagnosis: Epithelial ovarian cancers diagnosed in Norway 2004-2018 | | | | | | |
| --- | --- | --- | --- | --- | --- | --- |
| **Timing of exposure and exposure level** | Deaths | Person years | HR^a^ | 95% CI | | |
| **Pre-diagnosis** |  |  |  |  |  |  |
| No aspirin | 1982 | 15088 | Ref. |  |  |  |
| Aspirin use | 224 | 1210 | 1.04 | 0.89 | - | 1.20 |
|  |  |  |  |  |  |  |
| **Post-diagnosis, Baseline exposure** | | |  |  |  |  |
| No aspirin | 2056 | 15431 | Ref. |  |  |  |
| Aspirin use | 150 | 867 | 1.01 | 0.84 | - | 1.20 |
| Only post | 63 | 341 | 1.07 | 0.82 | - | 1.38 |
| Pre and post | 87 | 526 | 0.97 | 0.77 | - | 1.21 |
|  |  |  |  |  |  |  |
| **Post-diagnosis, Updated exposure** | | |  |  |  |  |
| No aspirin | 1969 | 14336 | Ref. | - |  | - |
| Ever aspirin | 237 | 1968 | 0.82 | 0.71 | - | 0.95 |
| Current aspirin | 174 | 1663 | 0.71 | 0.6 | - | 0.83 |
| Past aspirin | 63 | 305 | 1.50 | 1.16 | - | 1.95 |
|  |  |  |  |  |  |  |
| No aspirin | 1969 | 14336 | Ref. | - |  | - |
| DDD < median | 154 | 944 | 0.88 | 0.74 | - | 1.04 |
| DDD ≥ median | 83 | 1024 | 0.71 | 0.56 | - | 0.90 |
| ^a^Multivariable models controlling for age at diagnosis (continuous), histology groups (; high grade serous, low grade serous, endometrioid, mucinous, clear cell, carcinosarcoma), stage (localized, regional, distant, missing), ethnicity (Norway, other Nordic, other), education (mandatory level, secondary, higher education, missing), marital status (single, married/partnered, widowed/separated/divorced), and use of other medications at baseline (medications indicated for cardiovascular disease, and statins and anti-diabetics)  Abbreviations: DDD = defined daily dose; HR = hazard ratio | | | | | | |

| **Supplemental Table 4.** Pre- and post-diagnosis non-aspirin non-steroidal anti-inflammatory drug (NA-NSAID) use and overall survival following an ovarian cancer diagnosis: Epithelial ovarian cancers diagnosed in Norway 2004-2018 | | | | | | |
| --- | --- | --- | --- | --- | --- | --- |
| **Timing of exposure and exposure level** | **Deaths** | **Person years** | **HR^a^** | **95% CI** | | |
| **Pre-diagnosis** |  |  |  |  |  |  |
| No NA-NSAIDS | 2030 | 15068 | Ref. |  |  |  |
| NA-NSAIDS use | 176 | 1230 | 1.07 | 0.91 | - | 1.25 |
|  |  |  |  |  |  |  |
| **Post-diagnosis, Baseline exposure** | | |  |  |  |  |
| No NA-NSAIDS | 1943 | 14452 | Ref. |  |  |  |
| NA-NSAIDS use | 263 | 1846 | 1.08 | 0.92 | - | 1.27 |
| Only post | 203 | 1386 | 1.10 | 0.93 | - | 1.31 |
| Pre and post | 60 | 460 | 1.02 | 0.77 | - | 1.34 |
|  |  |  |  |  |  |  |
| **Post-diagnosis, Updated exposure** | | |  |  |  |  |
| No NA-NSAIDS | 1662 | 11741 | Ref. |  |  |  |
| Ever NA-NSAIDs | 544 | 4563 | 1.07 | 0.96 | - | 1.21 |
| Current NA-NSAIDs | 103 | 944 | 0.87 | 0.70 | - | 1.07 |
| Past NA-NSAIDs | 441 | 3619 | 1.14 | 1.00 | - | 1.29 |
|  |  |  |  |  |  |  |
| No NA-NSAIDS | 1662 | 11741 | Ref. |  |  |  |
| DDD < median | 279 | 2451 | 0.99 | 0.86 |  | 1.14 |
| DDD ≥ median | 265 | 2111 | 1.18 | 1.02 |  | 1.37 |
| ^a^Multivariable models controlling for age at diagnosis (continuous), histology groups (high grade serous, low grade serous, endometrioid, mucinous, clear cell, carcinosarcoma), stage (localized, regional, distant, missing), ethnicity (Norway, other Nordic, other), education (mandatory level, secondary, higher education, missing), marital status (single, married/partnered, widowed/separated/divorced), and use of other medications at baseline (medications indicated for cardiovascular disease, and statins and anti-diabetics)  Abbreviations: DDD = defined daily dose; HR = hazard ratio | | | | | | |

| **Supplemental Table 5.** Post-diagnosis low-dose aspirin and non-aspirin non-steroidal anti-inflammatory drug (NA-NSAID) use and survival following an ovarian cancer diagnosis defining post-diagnosis baseline exposure as minimum one or two prescriptions: Epithelial ovarian cancers diagnosed in Norway 2004-2018 | | | | | | |
| --- | --- | --- | --- | --- | --- | --- |
| **Timing of exposure and exposure level** | **Deaths** | **Person years** | **HR^a^** | **95% CI** | | |
| **Post-diagnostic use, minimum 1 prescription to be exposed** | | | | |  |  |
| No aspirin | 1696 | 14789 | Ref. |  |  |  |
| Aspirin use | 277 | 1882 | 0.96 | 0.83 | - | 1.11 |
| Only post | 120 | 616 | 0.94 | 0.77 | - | 1.14 |
| Pre and post | 157 | 1053 | 0.98 | 0.82 | - | 1.17 |
|  |  |  |  |  |  |  |
| No NA-NSAIDS | 841 | 6958 | Ref. |  |  |  |
| NA-NSAIDS use | 1132 | 9340 | 0.92 | 0.82 | - | 1.02 |
| Only post | 1018 | 8383 | 0.91 | 0.82 | - | 1.02 |
| Pre and post | 114 | 957 | 0.93 | 0.75 | - | 1.15 |
|  |  |  |  |  |  |  |
| **Post-diagnostic use, minimum 2 prescriptions to be exposed** | | | | |  |  |
| No aspirin | 1746 | 14789 | Ref. |  |  |  |
| Aspirin use | 227 | 1509 | 0.99 | 0.85 | - | 1.15 |
| Only post | 92 | 616 | 0.94 | 0.75 | - | 1.16 |
| Pre and post | 135 | 893 | 1.03 | 0.85 | - | 1.24 |
|  |  |  |  |  |  |  |
| No NA-NSAIDS | 1433 | 12086 | Ref. |  |  |  |
| NA-NSAIDS use | 540 | 4212 | 1.03 | 0.91 | - | 1.16 |
| Only post | 454 | 3476 | 1.03 | 0.91 | - | 1.16 |
| Pre and post | 86 | 736 | 1.02 | 0.81 | - | 1.28 |
| ^a^Multivariable models controlling for age at diagnosis (continuous), histology groups (high grade serous, low grade serous, endometrioid, mucinous, clear cell, carcinosarcoma), stage (localized, regional, distant, missing), ethnicity (Norway, other Nordic, other), education (mandatory level, secondary, higher education, missing), marital status (single, married/partnered, widowed/separated/divorced), and use of other medications at baseline (medications indicated for cardiovascular disease, and statins and anti-diabetics)  Abbreviations: DDD = defined daily dose; HR = hazard ratio | | | | | | |

| **Supplemental Table 6.** Post-diagnosis low-dose aspirin and non-aspirin non-steroidal anti-inflammatory drug (NA-NSAID) use and survival following an ovarian cancer diagnosis with updated exposure and two-years lagged exposure update: Epithelial ovarian cancers diagnosed in Norway 2004-2018 | | | | | | |
| --- | --- | --- | --- | --- | --- | --- |
|  | **Deaths** | **Person years** | **HR^a^** | **95% CI** | | |
| **Low-dose aspirin use** |  |  |  |  |  |  |
| No aspirin | 1087 | 10300 | Ref. |  |  |  |
| Ever aspirin | 96 | 1360 | 0.78 | 0.62 | - | 0.98 |
| Current aspirin | 79 | 1177 | 0.74 | 0.58 | - | 0.94 |
| Past aspirin | 17 | 183 | 1.17 | 0.72 | - | 1.91 |
|  |  |  |  |  |  |  |
| No aspirin | 1087 | 10300 | Ref. |  |  |  |
| DDD < median | 75 | 759 | 0.85 | 0.67 | - | 1.09 |
| DDD ≥ median | 21 | 601 | 0.58 | 0.37 | - | 0.92 |
|  |  |  |  |  |  |  |
| **NA-NSAID use** |  |  |  |  |  |  |
| No NA-NSAIDS | 898 | 8405 | Ref. |  |  |  |
| Ever NA-NSAIDs | 285 | 3254 | 1.11 | 0.94 | - | 1.30 |
| Current NA-NSAIDs | 42 | 679 | 0.66 | 0.48 | - | 0.90 |
| Past NA-NSAIDs | 243 | 2576 | 1.27 | 1.07 | - | 1.51 |
|  |  |  |  |  |  |  |
| No NA-NSAIDS | 898 | 8405 | Ref. |  |  |  |
| DDD < median | 154 | 1669 | 1.09 | 0.90 | - | 1.31 |
| DDD ≥ median | 131 | 1585 | 1.12 | 0.91 | - | 1.39 |
| ^a^Multivariable models controlling for age at diagnosis (continuous), histology groups (high grade serous, low grade serous, endometrioid, mucinous, clear cell, carcinosarcoma), stage (localized, regional, distant, missing), ethnicity (Norway, other Nordic, other), education (mandatory level, secondary, higher education, missing), marital status (single, married/partnered, widowed/separated/divorced), and use of other medications at baseline (medications indicated for cardiovascular disease, and statins and anti-diabetics)  Abbreviations: DDD = defined daily dose; HR = hazard ratio | | | | | | |

| **Supplemental Table 7.** Pre- and post-diagnosis low-dose aspirin use and survival following an ovarian cancer diagnosis for individuals with high-grade serous or metastatic disease at diagnosis: Epithelial ovarian cancers diagnosed in Norway 2004-2018 | | | | | | |
| --- | --- | --- | --- | --- | --- | --- |
| **Timing of exposure and exposure level** | **Deaths** | **Person-years** | **HR^a^** | **95% CI** | | |
| **Serous high-grade** |  |  |  |  |  |  |
| **Pre-diagnosis** |  |  |  |  |  |  |
| No aspirin | 1101 | 7366 | Ref. |  |  |  |
| Aspirin use | 99 | 592 | 1.05 | 0.84 | - | 1.30 |
|  |  |  |  |  |  |  |
| **Post-diagnosis, Baseline exposure** | | |  |  |  |  |
| No aspirin | 1132 | 7540 | Ref. |  |  |  |
| Aspirin use | 68 | 417 | 0.98 | 0.76 | - | 1.28 |
| Only post | 27 | 176 | 0.95 | 0.64 | - | 1.41 |
| Pre and post | 41 | 241 | 1.01 | 0.73 | - | 1.40 |
|  |  |  |  |  |  |  |
| **Post-diagnosis, Updated exposure** | | |  |  |  |  |
| No aspirin | 1092 | 6924 | Ref. |  |  |  |
| Ever aspirin | 108 | 1036 | 0.73 | 0.59 | - | 0.91 |
| Current aspirin | 82 | 870 | 0.66 | 0.52 | - | 0.83 |
| Past aspirin | 26 | 166 | 1.18 | 0.79 | - | 1.76 |
|  |  |  |  |  |  |  |
| No aspirin | 1092 | 6924 | Ref. |  |  |  |
| DDD below median | 69 | 501 | 0.76 | 0.59 | - | 0.98 |
| DDD above median | 39 | 536 | 0.67 | 0.48 | - | 0.94 |
|  | | | | | | |
| **Metastatic** |  |  |  |  |  |  |
| **Pre-diagnosis** |  |  |  |  |  |  |
| No aspirin | 1633 | 8904 | Ref. |  |  |  |
| Aspirin use | 182 | 803 | 1.08 | 0.92 | - | 1.27 |
|  |  |  |  |  |  |  |
| **Post-diagnosis, Baseline exposure** | | |  |  |  |  |
| No aspirin | 1700 | 9161 | Ref. |  |  |  |
| Aspirin use | 115 | 545 | 1.00 | 0.82 | - | 1.23 |
| Only post | 44 | 219 | 0.96 | 0.70 | - | 1.30 |
| Pre and post | 71 | 326 | 1.03 | 0.80 | - | 1.32 |
|  |  |  |  |  |  |  |
| **Post-diagnosis, Updated exposure** | | |  |  |  |  |
| No aspirin | 1657 | 8607 | Ref. |  |  |  |
| Ever aspirin | 158 | 1103 | 0.72 | 0.61 | - | 0.86 |
| Current aspirin | 118 | 930 | 0.63 | 0.52 | - | 0.77 |
| Past aspirin | 40 | 173 | 1.31 | 0.95 | - | 1.80 |
|  |  |  |  |  |  |  |
| No aspirin | 1657 | 8607 | Ref. |  |  |  |
| DDD below median | 114 | 582 | 0.81 | 0.66 | - | 0.99 |
| DDD above median | 44 | 521 | 0.55 | 0.40 | - | 0.75 |
| ^a^Multivariable models controlling for age at diagnosis (continuous), histology groups (for models including metastatic cases; high grade serous, low grade serous, endometrioid, mucinous, clear cell, carcinosarcoma), stage (for models including high grade serous; localized, regional, distant, missing), ethnicity (Norway, other Nordic, other), education (mandatory level, secondary, higher education, missing), marital status (single, married/partnered, widowed/separated/divorced), and use of other medications at baseline (medications indicated for cardiovascular disease, and statins and anti-diabetics)  Abbreviations: DDD = defined daily dose; HR = hazard ratio | | | | | | |

| **Supplemental Table 8.** Pre- and post-diagnosis non-aspirin non-steroidal anti-inflammatory drug (NA-NSAID) use and survival following an ovarian cancer diagnosis for individuals with high-grade serous or metastatic disease at diagnosis: Epithelial ovarian cancers diagnosed in Norway 2004-2018 | | | | | | |
| --- | --- | --- | --- | --- | --- | --- |
| **Timing of exposure and exposure level** | **Deaths** | **Person-years** | **HR^a^** | **95% CI** | | |
| **Serous high-grade** |  |  |  |  |  |  |
| **Pre-diagnosis** |  |  |  |  |  |  |
| No NA-NSAIDS | 1108 | 7398 | Ref. |  |  |  |
| NA-NSAIDS use | 92 | 560 | 1.12 | 0.90 | - | 1.39 |
|  |  |  |  |  |  |  |
| **Post-diagnosis, Baseline exposure** | | |  |  |  |  |
| No NA-NSAIDS | 1051 | 6929 | Ref. |  |  |  |
| NA-NSAIDS use | 149 | 1029 | 1.02 | 0.83 | - | 1.27 |
| Only post | 123 | 803 | 1.06 | 0.84 | - | 1.33 |
| Pre and post | 26 | 226 | 0.90 | 0.60 | - | 1.35 |
|  |  |  |  |  |  |  |
| **Post-diagnosis, Updated exposure** | | |  |  |  |  |
| No NA-NSAIDS | 878 | 5565 | Ref. |  |  |  |
| Ever NA-NSAIDs | 322 | 2395 | 1.11 | 0.95 | - | 1.3 |
| Current NA-NSAIDs | 59 | 490 | 0.92 | 0.70 | - | 1.21 |
| Past NA-NSAIDs | 263 | 1905 | 1.17 | 0.99 | - | 1.38 |
|  |  |  |  |  |  |  |
| No NS-NSAIDs | 959 | 5565 | Ref. |  |  |  |
| DDD below median | 186 | 1314 | 0.99 | 0.83 | - | 1.18 |
| DDD above median | 160 | 1081 | 1.12 | 0.92 | - | 1.35 |
|  |  |  |  |  |  |  |
| **Metastatic** |  |  |  |  |  |  |
| **Pre-diagnosis** |  |  |  |  |  |  |
| No NA-NSAIDS | 1672 | 9003 | Ref. |  |  |  |
| NA-NSAIDS use | 143 | 704 | 1.09 | 0.92 | - | 1.30 |
|  |  |  |  |  |  |  |
| **Post-diagnosis, Baseline exposure** | | |  |  |  |  |
| No NA-NSAIDS | 1594 | 8542 | Ref. |  |  |  |
| NA-NSAIDS use | 221 | 1164 | 1.14 | 0.96 | - | 1.36 |
| Only post | 173 | 894 | 1.16 | 0.96 | - | 1.41 |
| Pre and post | 48 | 270 | 1.09 | 0.80 | - | 1.48 |
|  |  |  |  |  |  |  |
| **Post-diagnosis, Updated exposure** | | |  |  |  |  |
| No NA-NSAIDS | 1373 | 7081 | Ref. |  |  |  |
| Ever NA-NSAIDs | 442 | 2629 | 1.14 | 1.01 | - | 1.30 |
| Current NA-NSAIDs | 88 | 610 | 0.93 | 0.74 | - | 1.16 |
| Past NA-NSAIDs | 354 | 2019 | 1.22 | 1.06 | - | 1.40 |
|  |  |  |  |  |  |  |
| No NS-NSAIDs | 1487 | 7081 | Ref. |  |  |  |
| DDD below median | 252 | 1410 | 1.04 | 0.89 | - | 1.20 |
| DDD above median | 224 | 1219 | 1.20 | 1.02 | - | 1.41 |
| ^a^Multivariable models controlling for age at diagnosis (continuous), histology groups (for models including metastatic cases; high grade serous, low grade serous, endometrioid, mucinous, clear cell, carcinosarcoma), stage (for models including high grade serious; localized, regional, distant, missing), ethnicity (Norway, other Nordic, other), education (mandatory level, secondary, higher education, missing), marital status (single, married/partnered, widowed/separated/divorced), and use of other medications at baseline (medications indicated for cardiovascular disease, and statins and anti-diabetics)  Abbreviations: DDD = defined daily dose; HR = hazard ratio | | | | | | |
